# Diode laser transscleral cyclophotocoagulation followed by phacotrabeculectomy on medically unresponsive acute primary angle closure eyes: the long-term result

**DOI:** 10.1186/1471-2415-14-26

**Published:** 2014-03-10

**Authors:** Wei Liu, Yanhui Chen, Yingjuan Lv, Linni Wang, Xiaoli Xing, Aihua Liu, Jian Ji

**Affiliations:** 1 Glaucoma department, Tianjin Medical University Eye Hospital, 251 Fukang Road, Nankai District, Tianjin, 300384, China

**Keywords:** Diode laser transscleral cyclophotocoagulation, Acute primary angle closure, Phacotrabeculectomy

## Abstract

**Background:**

To explore the intraocular pressure-lowering effect and complications of diode laser transscleral cyclophotocoagulation (DLTSC) followed by phacotrabeculectomy on medically unresponsive acute primary angle closure eyes.

**Methods:**

Nine eyes of nine medically unresponsive acute primary angle closure patients were enrolled. All the patients underwent cyclophotocoagulation followed by phacotrabeculectomy to control the prolonged acute attack. Data were recorded prospectively and then analyzed retrospectively. The reduction in intraocular pressure, improvement of vision and the complications were evaluated.

**Results:**

After DLTSC, the IOP of all the patients were reduced, but all were above 21 mmHg under topical anti-glaucoma medications. After phacotrabeculectomy, the IOP of all the patients was decreased. At the final visit, the vision of all the patients was improved and the IOP of all the patients was below 21 mmHg without anti-glaucoma medications. There were no complications during the DLTSC and phacotrabeculectomy. Uveitis was the common complications after the both procedures, which were resolved by medication treatment.

**Conclusion:**

Diode laser transscleral cyclophotocoagulation followed by phacotrabeculectomy is an alternative procedure to control the intraocular pressure of medically unresponsive acute primary angle closure eyes with few complications.

## Background

Acute primary angle closure (APAC) is a common ophthalmic emergency that requires early treatment. The longer the duration of increased intraocular pressure (IOP), the more irreversible damage there is to the optic nerve head, iris, lens, endothelium, and drainage pathways [1,2]. Early aggressive management of an acute episode is crucial to prevent development of primary angle closure glaucoma (PACG) after APAC and to relieve the excruciating symptoms of the patients [3].

The conventional treatment for APAC is to reduce IOP medically, after which laser peripheral iridoplasty or iridotomy (LPI) is performed. However, in some eyes, the acute attack is severe and often refractory to medical treatment [4-7], and the prolonged poor cornea clarity of APAC patients limits the application of LPI. In such cases, trabeculectomy is occasionally performed as an urgent intervention [6]. However, for medically unresponsive APAC cases, trabeculectomy on inflamed ‘hot’ eyes with high IOP poses a high risk of surgical failure and complications, such as suprachoroidal hemorrhage [4,8-10].

Is there any other method to control the high IOP of medically unresponsive APAC eyes? Diode laser transscleral cyclophotocoagulation (DLTSC), initially reserved for eyes with refractory glaucoma and limited visual prognosis, is now being used more widely, even as primary surgical treatment in glaucoma therapy, including primary open-angle and pseudoexfoliative glaucoma [7,11], chronic angle closure glaucoma [12,13], and even eyes with good vision [14]. In this study, we aimed to explore the safety and efficacy of DLTSC followed by phacotrabeculectomy on Chinese medically unresponsive APAC eyes.

## Methods

### Patients

Consecutive medically unresponsive APAC patients accepted DLTSC followed by phacotrabeculectomy from November 2008 to October 2010 were included in this study. Data were recorded prospectively and then analyzed retrospectively. This study adheres to the Declaration of Helsinki and was approved by the ethics committee of Tianjin Medical University Eye Hospital. Informed consent was obtained from each patient.

All the patients underwent a thorough ocular examination, including visual acuity, slit-lamp biomicroscopy, IOP, B-scan, ultrasound biomicroscopy etc at presentation. Post-operative evaluation was conducted on days 1, 7 and months 1, 3, 6 and 12. The patients, who needed closer follow-up, according to the clinical outcome, were evaluated monthly or even weekly. On each occasion, the patients were fully examined under the slit-lamp biomicroscopy. The visual acuity and IOP were determined. Any complications of surgery were noted. All the IOP was measured by non-contact tonometer (Full Auto Tonometer TX-F, Canon, Japan) for three times and the average was recorded.

### Diode laser transscleral cyclophotocoagulation

Diode laser transscleral cyclophotocoagulation was performed with the OcuLight SLx 810 nm diode laser photocoagulator and the handheld fiberoptic G-probe, both from Iris Medical Instruments (Mountain View, CA) [13,15]. All the treatments were performed under local anesthesia (2 ml of 2% lidocaine as a retrobulbar injection). The laser was set at an initial power of 1700 mW and a duration of 1 second. The laser power was titrated upwards and downwards during the procedure. The target was to achieve a ‘burst’ sound in roughly half of the laser applications. Laser applications were spaced evenly over the inferior 180 degrees, while sparing the 3- and 9-o’clock regions.

Post-operatively, all preoperative IOP-lowering medications, except systemic medications (such as high-osmotic agents) and topical pilocarpine, were continued. The patients were also given tobramycin and dexamethasone eye drops (TobraDex, S.A. Alcon-Couvreur N.V., Rijksweg, Puurs, Belgium) 6 times per day, 1% atropine gel (Dishan, Xingqi Co., Ltd, Shenyang, China) twice per day, and pranoprofen eye drops (Pranopulin, Senju Pharmaceutical Co., Ltd, Osaka, Japan) 4 times per day.

### Phacotrabeculectomy

All the surgeries were performed by the same experienced surgeon (JJ). During the surgery, after the fornix-based conjunctival flap and rectangular scleral flap were performed, mitomycin-C (MMC) was applied under the conjunctival and scleral flaps. Then, the phacoemulsification and intraocular lens (IOL) implantation was performed, followed by trabeculectomy and peripheral iridectomy. The scleral flap and the conjunctival wound were closed with 10/0 nylon sutures at the end of the surgery.

After surgery, all IOP-lowering medications were discontinued. Postoperative medications employed routinely are tobramycin and dexamethasone eye drops (TobraDex, S.A. Alcon-Couvreur N.V., Rijksweg, Puurs, Belgium) 6 times per day, and pranoprofen eye drops (Pranopulin, Senju Pharmaceutical Co., Ltd, Osaka, Japan) 4 times per day. All the drops were discontinued after 6 weeks of treatment.

## Results

### Patients’ characteristics

Nine consecutive medically unresponsive APAC patients (9 eyes) were enrolled in this study. All of the patients presented with symptoms of eye pain, blurred vision, headache and nausea. The duration of the acute attack ranged from 6 days to 23 days. The vision of the patients was threatened mostly by significant cornea edema. The IOP of the patients ranged from 43.2 mmHg to 58.7 mmHg, despite maximum medication. None of the patients were performed laser peripheral iridotomy for corneal edema. The detailed patient characteristics are indicated in Table 1.

**Table 1:** The patients’ characteristics

**Case**	**Eye affected**	**Durations (days)**	**Medical treatment before cyclophotocoagulation**	**Presenting IOP (mmHg)**	**VA (LogMAR)**
1	Left	7	1% pilocarpine QID, 2% carteolol Q12H, 0.2% Brimonidine BID	43.2	HM
2	Left	11	1% pilocarpine Q2H, 1% Brinzolamide TID, 0.2% Brimonidine BID, oral Methazolamide 25 mg BID, intravenous 20% mannitol 250 ml QD	43.6	1.0
3	Right	7	1% pilocarpine QID, 2% carteolol Q12H, 0.2% Brimonidine BID, intravenous 20% mannitol 400 ml QD	45.0	2.0
4	Left	6	1% pilocarpine QID, 2% carteolol Q12H, 1% Brinzolamide TID, oral Methazolamide 50 mg BID, intravenous 20% mannitol 400 ml Q12H	48.6	0.9
5	Right	23	1% pilocarpine QID, 2% carteolol Q12H, 1% Brinzolamide TID, oral Methazolamide 25 mg BID, intravenous 20% mannitol 250 ml Q12H	54.2	HM
6	Left	20	1% pilocarpine TID, 2% carteolol Q12H, 1% Brinzolamide TID, 0.2% Brimonidine BID, oral Methazolamide 50 mg BID	58.7	HM
7	Left	15	1% pilocarpine QID, 2% carteolol Q12H, 1% Brinzolamide TID, 0.2% Brimonidine BID, oral Methazolamide 25 mg BID	44.8	FC
8	Right	17	1% pilocarpine QID, 1% Brinzolamide TID, 0.2% Brimonidine BID, oral Methazolamide 25 mg BID, intravenous 20% mannitol 250 ml QD	47.0	1.0
9	Right	12	1% pilocarpine QID, 2% carteolol Q12H, 1% Brinzolamide TID, 0.2% Brimonidine BID, oral Methazolamide 25 mg BID, intravenous 20% mannitol 400 ml QD	50.0	FC

IOP: intraocular pressure, VA: vision acuity, HM: hand movement, FC: finger counting.

Case 1, 6 and 7 were not administered mannitol for systemic disease (such as renal dysfunction, cardiac insufficiency, etc), case 2 and 8 were not administered beta-blocker for atrioventricular block, case 1 and 3 were not administered carbonic anhydrase inhibitors for medication allergy.

### Clinical outcome after DLTSC

There were no complications during the DLTSC procedure. After DLTSC, the IOP of all the patients was lower, but all were above 21 mmHg (from 23.3 to 32 mmHg) under topical anti-glaucoma medications. After DLTSC, corneal edema of all the patients was relieved in part. All the patients experienced uveitis (aqueous flare: from ++ to +++), 5 patients experienced subconjunctival hemorrhage, and only 1 patient experienced exudation in the anterior chamber, indicating a relatively severe inflammatory reaction.

### Clinical outcome after phacotrabeculectomy

After phacotrabeculectomy, the IOP of all the patients was lower. The follow-up period ranged from 14 months to 35 months. At the final visit, the vision of all the patients was improved and the IOP of all the patients was below 21 mmHg (from 12.8 to 18 mmHg) without anti-glaucoma medications or other surgical interventions. The detailed IOP results are indicated in Table 2.

**Table 2:** Clinical outcome after DLTSC and phacotrabeculectomy

**Case**	**1**	**2**	**3**	**4**	**5**	**6**	**7**	**8**	**9**
Total laser energy (J)	68	100	68	90	110	76	82	90	86
IOP 1 day after laser (mmHg)	27.8	23.3	30.0	29.5	31.1	28.4	25.9	32.0	27.7
Days between procedures (depending on the corneal clarity and inflammation)	3	8	2	3	5	7	10	4	7
MMC (mg/ml, min)	0.2, 1.5	0.2, 1.0	0.2, 2.0	0.3, 2.0	0.2, 2.0	0.2, 3.0	0.2, 2.0	0.3, 2.0	0.2, 2.0
IOP before surgery (mmHg)	23.9	22.0	28.0	31.0	28.7	32.0	29.7	26.6	25.7
IOP 1 day after surgery (mmHg)	11.6	19.6	8.6	21.7	15.8	22.0	14.6	18.7	23.0
IOP 1 week after surgery (mmHg)	17.1	11.0	10.0	20.4	17.4	15.9	13.9	14.8	9.0
IOP 1 month after surgery (mmHg)	8.7	13.5	14.8	9.5	14.3	16.1	14.1	13.3	10.5
IOP 6 months after surgery (mmHg)	10.2	12.8	13.9	14.7	15.6	14.4	10.7	16.1	9.5
IOP 1 year after surgery (mmHg)	11.2	11.5	12.3	13.5	16.5	15.7	13.2	17.1	10.7
Final IOP (mmHg) (months from presentation)	13.4 (22)	14.2 (25)	15.6 (30)	12.8 (35)	14.3 (14)	16.1 (17)	12.9 (15)	18.0 (14)	13.1 (17)
Final VA (LogMAR)	0.7	1.0	0.5	0.4	0.3	0.5	0.2	0.2	0.3

DLTSC: diode laser transscleral cyclophotocoagulation, IOP: intraocular pressure, MMC: mitomycin-C, VA: vision acuity.

There were no complications during the surgery. At 1 day after surgery, all the patients presented with cornea edema and uveitis and 3 patients presented with exudation in the anterior chamber, which were resolved within a week by medication treatment alone (2 patients) or combined with YAG laser (1 patient). No other complications were encountered during the follow-up after the surgery.

## Discussion

To date, many methods have been studied to control the IOP of APAC. The conventional initial treatment may involve one or more of the IOP-lowering drugs. However, for a significant proportion of APAC patients, these treatments may fail to reduce IOP. [1] Laser peripheral iridotomy appears to be effective and safe in controlling rising IOP [16-19]. However, for Asian eyes, the results after LPI are not satisfactory [20,21]. Moreover, the application of LPI is compromised by the edematous cornea of APAC patients. In cases when LPI is not possible, immediate paracentesis has been proposed as an alternative procedure to rapidly lower the IOP [22]. However, the performance of paracentesis in an eye with APAC has many unique technical difficulties [1], and the paracentesis itself runs the risk of ocular decompression retinopathy [23] and intraocular inflammation. Therefore, in selected cases, surgical intervention may need to be considered as a primary procedure to lower the IOP [6]. However, for medically unresponsive cases of APAC, trabeculectomy on eyes with high IOP has a high risk of surgical failure and complications, especially suprachoroidal hemorrhage [4]. As a severely debilitating complication of glaucoma surgery, suprachoroidal hemorrhage has been reported to be strongly associated with the level of the preoperative IOP in several studies [8-10].

Diode laser transscleral cyclophotocoagulation, traditionally used only in eyes with advanced end stage glaucoma and with little or no visual potential, has now become a popular minimally invasive treatment for glaucoma [7,11-14]. The 810 nm diode wavelength is twice as efficient for DLTSC than the Nd:YAG wavelength of 1064 nm, and the near infrared light generated by the diode laser is absorbed better by melanin than the longer wavelengths [14,24]. This advantage allows the diode laser to use less energy to produce comparable lesions.

In this study, for medically unresponsive APAC eyes, firstly, we tried DLTSC to control the high IOP. Although the IOP was decreased after DLTSC, the desired degree of reduction was not achieved. The authors think this is mostly because of the conservative protocol for DLTSC in our study, or APAC is not sensitive to DLTSC, which needs further study to confirm. In order to avoid severe complication, we adopted the narrow laser scope (only the inferior 180 degrees), a short laser duration time (1000 ms) and low laser energy (1700 mW) during the DLTSC. The only article covering the use of DLTSC on APAC eyes in the literature included 5 APAC cases from two ophthalmic units in the United Kingdom [25]. All of their 5 cases achieved successful control of IOP and resolution of the acute attack after DLTSC, which is different from the results in our case series. The mechanism of their better control of IOP was not clear; we think it may be partly due to the iridotomy or iridoplasty performed before DLTSC in most of their cases.

Although the IOP was not controlled under 21 mmHg after DLTSC, the cornea edema was relieved and the IOP was decreased in part, which enabled us to perform phacotrabeculectomy with a lower risk of severe complications. After the surgery, the IOP of all the eyes was controlled to an ideal level and visual acuity was significantly improved. Moreover, except for uveitis and cornea edema, no other severe complications (such as vision loss, phthisis and suprachoroidal hemorrhage, etc.) were encountered after DLTSC and filtration surgery. One reason might be the comparatively low laser energies we applied in comparison with other investigators, who treated with 1.75–3 W for 2 s [26-29]. Another might be the relieved corneal edema and the decreased IOP by DLTSC before phacotrabeculectomy, which simplified the surgical manipulations.

The authors acknowledge the limitations of the study. This was a non-comparative case series and the sample size was relatively small. The laser energy employed and the MMC protocol used were not uniform and the IOP-lowering effect of cataract surgery alone was not evaluated. A prospective comparative study (filtration surgery after DLTSC versus filtration surgery under high IOP) with larger samples would be ideal. Further study to compare the IOP-lowering effect of different DLTSC protocols on APAC eyes is needed. However, in this study, the authors did, indeed, propose an alternative procedure to control the IOP of medically unresponsive APAC eyes without severe complications.

## Conclusions

Diode laser transscleral cyclophotocoagulation followed by phacotrabeculectomy is an alternative procedure to control the intraocular pressure of medically unresponsive acute primary angle closure eyes with few complications.

## Competing interests

The authors declare that they have no competing interests.

## Authors’ contributions

JJ and XX designed and supervised the study; LW drafted the manuscript; CY and WL collected the data; LY and LA analyzed the data and helped to draft the manuscript. All authors read and approved the final manuscript.

## Pre-publication history

The pre-publication history for this paper can be accessed here:

http://www.biomedcentral.com/1471-2415/14/26/prepub
